# Can the Combination of Rehabilitation and Vitamin D Supplementation Improve Fibromyalgia Symptoms at All Ages?

**DOI:** 10.3390/jfmk7020051

**Published:** 2022-06-20

**Authors:** Dalila Scaturro, Fabio Vitagliani, Sofia Tomasello, Mirko Filippetti, Alessandro Picelli, Nicola Smania, Giulia Letizia Mauro

**Affiliations:** 1 Department of Surgical, Oncological and Stomatological Disciplines, University of Palermo, 90127 Palermo, Italy; giulia.letiziamauro@unipa.it; 2 Faculty of Medicine and Surgery, University of Catania, 90121 Catania, Italy; fabiovitagliani93@libero.it; 3 Faculty of Medicine and Surgery, University of Palermo, 90127 Palermo, Italy; sofiatomasello@alice.it; 4 Neurorehabilitation Unit, Department of Neurosciences, University Hospital of Verona, 37100 Verona, Italy; mirko.filippetti@univr.it (M.F.); alessandro.picelli@univr.it (A.P.); nicola.smania@univr.it (N.S.); 5 Neuromotor and Cognitive Rehabilitation Research Center, Physical and Rehabilitation Medicine Section, Department of Neurosciences, Biomedicine and Movement Sciences, University of Verona, 37100 Verona, Italy

**Keywords:** fibromyalgia, vitamin D, rehabilitation, musculoskeletal diseases

## Abstract

Several studies have indicated a correlation between vitamin D deficiency and widespread chronic pain syndromes, such as fibromyalgia. During this study, the effect of supplementation with vitamin D in association with physical exercise in patients with fibromyalgia was evaluated, in terms of improvement of pain, functional capacity and quality of life, also evaluating the presence of any differences in age. A single-center, observational, comparative study was conducted in 80 fibromyalgia patients. They are randomized into 2 groups: Group A, consisting of patients ≤50 years; and group B, consisting of patients >50 years. Both received weekly supplementation with 50,000 IU cholecalciferol for 3 months in association with a rehabilitation protocol. Patients were assessed at enrollment (T0), 3 months (T1), and 6 months (T2) from the initial assessment with blood vitamin D dosage and administration of rating scales (NRS, FIQ, and SF-12). From the comparison between the two groups, we have seen that in young people, supplementation with high-dose vitamin D improves short-term musculoskeletal pain and long-term functional capacity. Conversely, musculoskeletal pain and long-term quality of life improve in the elderly. Supplementing with high doses of vitamin D in fibromyalgia patients improves the quality of life and pain in the elderly and also the functional capacity in the young.

## 1. Introduction

Fibromyalgia, according to the American College of Rheumatology (ACR), is a chronic pain syndrome characterized by generalized musculoskeletal pain lasting more than 3 months and acupressure hyperalgesia in particular points defined as “tender points” [1,2]. Affected patients, in addition to pain, often also have associated psychosomatic symptoms, such as sleep disturbances, cognitive disturbances, fatigue, anxiety and/or depression, headache, irritable bowel, and abdominal pain [3,4,5].

It is the second most common rheumatological disorder, after arthrosis, with a higher prevalence between the ages of 20 and 50 [5]. Its prevalence is 3–6% in the general population [6], with a male/female ratio of 1:9 [7]. The pathology is very complex and heterogeneous which causes an important functional limitation in patients, negatively affecting their quality of life and favoring social isolation and an increase in healthcare costs [8,9].

Its etiology to date always remains unknown. Several authors speculate that neurotransmitter abnormalities and endocrine system disorders are involved in its genesis. This gives rise to a central sensitization syndrome with dysfunction of the neurocircuits responsible for the perception, transmission, and processing of nociceptive afferents, with a prevalent manifestation of pain in the musculoskeletal system [10,11,12].

Numerous other genetic and/or hormonal factors are likely related to the genesis of this disease. Among these, several studies have indicated a correlation between vitamin D deficiency and widespread chronic pain syndromes, such as fibromyalgia [10,13,14,15]. In patients with fibromyalgia, the presence of a picture of hypovitaminosis D is frequently found [16,17].

Vitamin D is a pleiotropic hormone, mainly derived from skin synthesis through ultraviolet radiation, which regulates bone metabolism [18]. It intervenes in the regulation of the immune system and also promotes the release of nerve and neurotrophic growth factors and influences the inflammatory processes responsible for the persistence of chronic pain [19].

Hypovitaminosis D generally manifests itself with symptoms almost overlapping with those of fibromyalgia, especially with fatigue and pain and widespread muscle weakness [18].

To date, there is no specific and effective treatment for the complete resolution of symptoms in patients with fibromyalgia [20]. Current treatment focuses essentially on symptom management and quality of life improvement [21]. According to the guidelines of the European League Against Rheumatism of 2017, the approach should be multidisciplinary and gradual, through: changes in lifestyle and behavior, physical exercise, and drug therapy [22].

Drug therapy makes use of drugs that act at the level of the central nervous system, such as antidepressants (e.g., Tricyclic antidepressants, SNRIs, SSRIs), muscle relaxants, and anticonvulsants. These drugs act at the level of neuromodulators involved in the pathogenesis of the disease, but are associated with the appearance of various side effects [21,23].

Exercise is the most suitable non-pharmacological treatment, but about the heterogeneity of the disease, it must be individualized based on the physical function of the patients, the severity of pain, and other symptoms [21]. The physical exercise recommend must include aerobic exercises; progressive resistance group exercise; muscle strengthening exercises with low workloads; and stretching exercises of the muscle groups of the upper and lower limbs, to recover elasticity and reduce muscle contractures. Relaxation techniques, decontracting massage therapy, hydrotherapy, and electrotherapy [21,24] are also very useful.

In addition, a proposed link between gut microbiome models and chronic pain syndromes has led to studies investigating probiotics as a possible treatment through nutrient supplementation. It is a highly studied treatment modality as numerous treatments have been linked to FM [25].

Despite its possible etiological role in the genesis of fibromyalgia, the effectiveness of supplementing with vitamin D in fibromyalgia patients remains questionable [17]. A randomized controlled clinical trial has shown the efficacy of supplementation with vitamin D in reducing the symptoms of pain and fatigue in affected patients [26]. However, another study found no correlation between vitamin D supplementation and improved health status [27].

During this study, the effect of supplementation with vitamin D in association with physical exercise in patients with fibromyalgia was evaluated, in terms of improvement of pain, functional capacity and quality of life, also evaluating the presence of any differences in age.

## 2. Materials and Methods

A comparative, observational, single-center study was conducted on afferent fibromyalgia patients at the U.O.C. of Recovery and Functional Rehabilitation of the A.O.U.P. “P. Giaccone” of Palermo. The study was conducted in the period between September 2019 and April 2021.

The study was conducted by the ethical guidelines of the Declaration of Helsinki; the local ethics committee “Palermo 1” approved the study, with the reference number 06/2019; the information and data were managed according to the guidelines of the Good Clinical Practice (GCP). All participants signed an informed consent form at the time of enrollment to collect clinical data.

The inclusion criteria were: age ≥ 18 years; diagnosis of fibromyalgia syndrome according to the 2013 revised criteria of the American College of Rheumatology (ACR) [4]; blood value of vitamin D ≤ 30 ng/mL; no previous or current drug treatment with vitamin D; written informed consent for participation and acceptance of the study. Blood samples were taken from patients in September during which the vitamin D level is the highest. Serum vitamin D 25-OH level was measured using an enzyme-linked immunosorbent assay (ELISA) method. Vitamin D values were classified as deficient (≤20 ng/mL), insufficient (21 to 30 ng/mL) and sufficient (31 to 60 ng/mL) [28].

The exclusion criteria were: osteoporosis, the presence of comorbidities, such as renal or hepatic insufficiency, which interfered with the absorption and/or metabolism of vitamin D; malabsorption syndromes; pregnancy and/or bed rest; and presence of other autoimmune, psychiatric, neoplastic and endocrine rheumatic diseases.

The recruited patients were randomized, based on their age, into 2 groups: Group A, comprising 20 patients aged ≤50 years; and group B, comprising 60 patients aged >50 years.

Both groups received supplementation with cholecalciferol 50,000 I.U. orally every week for 3 months in association with a rehabilitation protocol of 20 sessions.

The rehabilitation sessions were held three times a week with a duration of 100 min. Each session consisted of 40 min of aerobic exercises, 30 min of LaserCO_2_, and 15 min of TENS on specific tender points.

Aerobic exercises promote the loss of body weight and help reduce the work of the antigravity muscles, with a muscle relaxant and analgesic effect. The aerobic exercises consisted of three parts: the first part of warm-up lasting 10 min, consisting of breathing exercises, joint mobilization, and stretching performed at 70–80% of the maximum heart rate; a second part lasting 20 min consisted of high-intensity exercises, such as stretching, proprioceptive exercises, and neuromuscular coordination exercises; and a third part lasting 10 min designed to gradually lower the heart rate [29].

The low-energy laser (LaserCO_2_) is a widely used tool used in the treatment of musculoskeletal disorders, including fibromyalgia. It was applied to the three most painful points. It acts through an anti-inflammatory, anti-edema, and analgesic action, with short and long-term efficacy in the treatment of fibromyalgia [29].

TENS is a non-invasive method with analgesic action that is carried out through the excitation of the sensory nerves and the stimulation of the gate pain mechanism and/or the opioid system. It allows the reduction in pain and fatigue, improving hyperalgesia and functional capacity in patients with fibromyalgia [29].

All patients were allowed to continue taking personal drug therapy for fibromyalgia.

Patients were evaluated at the time of enrollment (T0), at 3 months (T1) and 6 months (T2) from the initial evaluation, through a specialist physiatric visit, medical history (age, sex, BMI, education level, occupation, and years from diagnosis), blood dosage of vitamin D and administration of evaluation scales, such as Numerical Rating Scale (NRS), Fibromyalgia Impact Questionnaire (FIQ), and Short Form 12 Health Survey (SF-12).

The NRS scale is a quantitative rating scale by which patients are asked to rate their pain on a defined scale, from 0 to 10, which is easy to use and has high validity and reliability coefficients [30].

The FIQ questionnaire consists of 10 questions. The first question contains 11 items relating to the ability in the last week to carry out activities of daily life, with a variable score between 0 (always) and 3 (never). The second and third questions ask the number of days in the last week that the patient felt well and was unable to carry out their work (including housework) due to symptoms of fibromyalgia. Questions from 4 to 10 are horizontal linear scales on which the patient evaluates the difficulty of work, pain, fatigue, morning fatigue, stiffness, anxiety, and depression. The maximum score of the FIQ is 100; in patients with fibromyalgia, the average values are around 50, while only patients with severe clinical pictures have results above 70 [31].

The 12-Item Short Form Survey (SF-12) scale is the abbreviated version of the Short Form 36 items Health Survey (SF36) questionnaire and serves as a generic indicator of the quality of life. It consists of 12 questions that investigate 8 different health aspects: physical activity, role limitations due to physical health, emotional state, physical pain, perception of general health, vitality, social activities, and mental health. The lower the score, the greater the degree of disability [32].

The primary endpoints assessed were: pain, using the NRS scale; and blood levels of vitamin D, by blood assay.

The secondary endpoints evaluated were: functional capacity, using the FIQ scale; and quality of life, using the SF-12 scale.

### Statistic Analysis

The data obtained were indexed on an Excel sheet and the subsequent statistical analysis was performed using the R software (R Foundation for Statistical Computing, Vienna, Austria).

The sample size was calculated with the formula below:

$$n=\frac{z_{\frac{\alpha }{2}}^{2}\sigma^{2}}{\varepsilon^{2}}=\frac{z_{\frac{\alpha }{2}}^{2}\pi \left(1-\pi \right)}{\varepsilon^{2}}$$

The type I error is equal to 0.05 (the quantile in the formula is equal to 1.96). The denominator *ε* = 0.13 is the maximum error acceptable to the researcher, and the choice of *ε* is arbitrary. Finally, we use the worst-case scenario of the proportion equal to 0.5. the formula gave a result equal to 56.83. Consequently, the number of 80 patients considered was sufficient to prove our thesis.

The descriptive analysis was performed based on the mean and standard deviation. For the statistical modeling we used the classic linear regression model to evaluate the effect net of any confounding variables. *p* values < 0.05 were considered statistically significant.

## 3. Results

In the course of this study, 250 subjects diagnosed with fibromyalgia were examined. Of these, 170 did not meet the criteria for inclusion in the study and were therefore excluded, because: 68 had comorbidities (neoplastic, rheumatic and/or psychiatric diseases), 2 were younger than 18 years, 53 had blood values of vitamin D > 30 ng/mL and 47 did not give informed consent to the study. Only 80 subjects were therefore included in the study. The baseline characteristics of the study participants are summarized in Table 1.

The 80 patients with fibromyalgia included in the study were aged between 34 and 70 years, with a mean age of 54.1 ± 9.1. 74 were female (92.5%) and only 6 were male (7.5%). The mean BMI was 27.4 ± 3.7 kg/m^2^. 38 patients (47.5%) had primary school education, 29 (36.2%) secondary school, and 13 (16.3%) had a university degree. 55 patients (68.7%) were employed, while the remaining 25 (31.3%) were unemployed. Patients had a mean pain duration of 2.8 ± 1.1 years.

At the initial clinical assessment (T0), the study participants’ blood vitamin D values averaged 22.8 ± 5.1 ng/mL. 19 (23.8%) had vitamin D values < 10 ng/mL; 21 (26.3%) had vitamin D values between 10 and 20 ng/mL; and 40 (49.9%) had vitamin D values > 20 ng/mL.

Study participants at T0 reported a mean pain value, calculated using the VAS scale, of 7.42 ± 0.86.

Finally, the initial average score of the FIQ-SCORE was 55.67 ± 7.68, while that of the SF12-Score was 25.77 ± 3.1. No statistically significant difference at baseline was observed between the two groups, except for age.

Table 2 and Table 3 show the effects of rehabilitation treatment and integration with cholecalciferol 25,000 IU in group A at 3 months (Table 2) and 6 months (Table 3).

In group A at T1, we observed statistically significant improvements regarding: disease-related pain (NRS: 7.6 ± 1.1 vs. 5.9 ± 1.1; *p* < 0.05); the score on the FIQ scale (58.1 ± 9.2 vs. 45.3 ± 6.6; *p* < 0.05); serum levels of vitamin D (22.3 ± 4.2 ng/mL vs. 31.1 ± 3.8 ng/mL; *p* <0.05; and quality of life (SF-12: 25.2 ± 3, 5 vs. 28.9 ± 2.7; *p* < 0.05) (Table 2). These statistically significant improvements were maintained even at T2, as regards: disease-related pain (NRS: 7.6 ± 1.1 vs. 5.7 ± 0.9; *p* < 0.05), the FIQ scale score (58.1 ± 9.2 vs. 44.4 ± 5.9; *p* < 0.05) and serum levels of vitamin D (22.3 ± 4.2 vs. 36.9 ± 4.1; *p* < 0.05). At 6 months the score on the SF-12 scale showed no statistically significant improvement (25.2 ± 3.5 vs. 27.2 ± 3.6; *p* = 0.08).

Table 4 and Table 5 show the effects of rehabilitation treatment and integration with cholecalciferol 25,000 IU in group B at 3 months (Table 4) and at 6 months (Table 5). At T1, statistically significant improvements were observed for: serum levels of vitamin D (23.1 ± 3.8 ng/mL vs. 33.3 ± 3.3 ng/mL; *p* < 0.05), related pain to the disease (NRS: 7.4 ± 0.8 vs. 6.1 ± 1.3; *p* < 0.05); FIQ scale score (54.8 ± 6.9 vs. 43.1 ± 7; *p* < 0.05); and quality of life (SF-12: 25.9 ± 2.9 vs. 30.3 ± 2.5; *p* < 0.05) (Table 4). At 6 months (T2) these statistically significant improvements were maintained for: pain related to the disease (7.4 ± 0.8 vs. 5 ± 1.1; *p* < 0.05); the serum levels of vitamin D (23.1 ± 3.8 vs. 39.2 ± 2.9; *p* < 0.05); and quality of life (SF-12: 25.9 ± 2.9 vs. 31.4 ± 2.4; *p* < 0.05). In group B at T2 we did not observe statistically significant improvements for the FIQ scale score (54.8 ± 6.9 vs. 52.4 ± 8.1; *p* = 0.08) (Table 5).

Finally, Table 6 shows the comparison between the T1-T0 (ΔT1-T0) and T2-T0 (ΔT2-T0) variations between the two groups at 3 months (T1) and at 6 months (T2). At T1, group A showed a statistically significant change in pain compared to group B (1.7 ± 0.8 vs. 1.3 ± 0.8; *p* = 0.03). No statistically significant difference between the two groups was instead observed for the vitamin D values (8.8 ± 2.7 vs. 10.2 ± 3.6; *p* = 0.12). FIQ scale scores (12.8 ± 4.8 vs. 11.8 ± 2.7; *p* = 0.25) and SF-12 (3.7 ± 1.8 vs. 4.4 ± 1.8; *p* = 0.14). At T2, group A showed greater statistically significant improvements for functional capacity (FIQ: 13.7 ± 2.7 vs. 2.4 ± 7.9; *p* < 0.05), compared to Group B. In group B instead the change in the score on the NRS scale was higher than in group A (2.4 ± 0.8 vs. 1.9 ± 0.8; *p* < 0.05), as was that of the SF-12 scale (5.5 ± 2.3 vs. 2 ± 3.5; *p* < 0.05). Finally, the variations in vitamin levels between the two groups did not show any statistically significant difference (14.6 ± 3.7 vs. 16.1 ± 3.5; *p* = 0.10).

## 4. Discussion

Fibromyalgia syndrome is a pathological condition that affects about 1.5–2 million people in Italy, with a significant clinical and economic impact [33]. Due to the complex clinical picture, it is estimated that an average of 7 years elapse between the onset of symptoms and the time of diagnosis of fibromyalgia [34].

Vitamin D deficiency is very common in patients with fibromyalgia and similarities have been observed between the symptoms of FMS and those of hypovitaminosis D. Despite this, even today the relationship between FMS and hypovitaminosis D remains controversial, just as conflicting are the data on the role of vitamin D supplementation in the management of fibromyalgia [35].

Several authors believe that serum vitamin D deficiency plays a role in the etiopathogenesis of fibromyalgia, indicating it as a possible risk factor. Vitamin D deficiency is related to several musculoskeletal disorders, as well as autoimmune diseases, metabolic syndrome, impaired cognitive function, psychiatric disorders, and increased risk of some cancers [36,37,38].

Vitamin D is a steroid hormone with action on various body tissues and organs, including the musculoskeletal system. Skeletal muscles possess vitamin D receptors and require it for their maximum function. Consequently, vitamin D deficiency results in muscle weakness and decreased muscle strength. Furthermore, the role of vitamin D in regulating the immune system, with the consequent increase in proinflammatory cytokines, may explain the origin of musculoskeletal pain related to vitamin D deficiency [39,40]. Based on this, it has been suggested the importance of supplementation with vitamin D in the treatment of rheumatological diseases, such as fibromyalgia, rheumatoid arthritis, and systemic lupus erythematosus precisely because of its role in regulating the immune system [41,42].

Lewis et al. instead affirm that FM causes hypovitaminosis D as a result of blocking the parathyroid axis, an endocrine system that activates vitamin D, suggesting that FM can suppress the production of vitamin D activated through the parathyroid axis and the secretion of phosphorus from part of the kidney [43].

In the light of the aforementioned hypotheses, the integration of vitamin D in patients with fibromyalgia appears rational to reduce pain and improve the quality of life. However, some authors suggest the lack of efficacy of supplementation with vitamin D in fibromyalgia patients [27,44,45,46]. Two controlled studies [47,48] found no correlation between low vitamin D levels and widespread musculoskeletal pain, as well as observed no significant improvement in musculoskeletal symptoms or quality of life after vitamin D supplementation.

In the course of our study, we evaluated the effectiveness of a combination of rehabilitation treatment and supplementation with vitamin D in short and long-term fibromyalgia patients, also trying to highlight any differences based on the different ages.

The novelty of our study was to show how supplementation with vitamin D is effective in fibromyalgia patients of any age. We observed that the weekly high-dose supplementation with short-term vitamin D resulted in marked improvements in pain, serum levels of vitamin D, and scores on the FIQ and SF-12 scale in both young and elderly patients. However, in the long term, these improvements were not maintained in quality of life in young patients and functional capacity in elderly patients.

Another important piece of data was extrapolated from the comparison between the two groups of patients. In young people, supplementation with vitamin D at high doses seems to significantly improve short-term musculoskeletal pain and long-term functional capacity. Conversely, significant improvements in musculoskeletal pain and long-term quality of life are observed in the elderly.

Our results are in line with most of the studies in the literature that have demonstrated the effectiveness of supplementation with vitamin D in improving the symptoms of fibromyalgia patients, even if there is disagreement on what the improvement parameters are [7,14,17,26,49,50].

Some authors [17,35] in their studies have observed that supplementing vitamin D in patients with fibromyalgia is a safe and economical treatment, with positive effects on the quality of life in patients, without however causing significant improvements in pain.

Bennet et al. showed a high frequency of vitamin D deficiency in patients with fibromyalgia, supporting the effectiveness of supplementing with vitamin D in terms of improving functional capacity and mood quality [49].

Wepner et al. concluded that patients who received supplementation with vitamin D, compared to placebo, had positive effects on pain and fatigue, without however showing improvements in terms of morning stiffness [26]. Furthermore, Matthana et al. showed that the improvement in painful symptoms in fibromyalgia patients was greater the higher the levels of vitamin D [50].

Finally, a meta-analysis of 12 observational studies revealed that vitamin D deficiency alone may not be sufficient to explain the symptom complex of fibromyalgia. However, the authors concluded that prolonged intake of vitamin D in fibromyalgia patients could still help reduce pain and improve anxiety, depression, and quality of life [14].

We believe that the correction of vitamin deficiency is a safe, low-cost, and effective therapeutic method in reducing the clinical symptoms of fibromyalgia in both young and elderly people, helping to improve pain, functional capacity, depression, and/or anxiety in these patients.

The main limitations of our study are represented primarily by the small sample size, resulting from the recruitment issues related to the SARS-CoV2 pandemic. A further limitation is the lack of a nutritional table of the subjects. It would have been an important finding as some authors suggest that low levels of magnesium and zinc promote excitotoxicity [51,52]. Another limitation is the lack of a control group that would have helped to strengthen our results. Finally, a further limitation is represented by the fact of having included in the study only the fibromyalgia patients who presented a picture of hypovitaminosis D.

## 5. Conclusions

There is common agreement in the literature that vitamin D may play a role in fibromyalgia, however, the type of relationship (cause or effect) is still an important subject of debate.

Despite this, based on our results, we recommend the introduction into clinical practice of the dosage of vitamin D and the correction of hypovitaminosis D in fibromyalgia patients with high weekly doses regardless of age.

This can contribute to the improvement of the clinical picture, in terms of improving the quality of life and pain in the elderly and also the functional capacity in the young.

However, further case-control studies are needed to further verify our hypotheses to be able to generalize our results.

## Figures and Tables

**Table 1:** General characteristics of patients at baseline.

Characteristics	Total (*n* = 80)	Group A (*n* = 20)	Group B (*n* = 60)	*p*-Value
Age (years), mean ± SD	54.1 ± 9.1	41.2 ± 6.1	58.3 ± 4.8	<0.05
Gender, *n* (%) Female Male	74 (92.5) 6 (7.5)	18 (90) 2 (10)	56 (93.3) 4 (6.7)	0.38
BMI, mean ± SD	27.4 ± 3.7	26.7 ± 3.1	27.2 ± 2.8	0.50
Level education, *n* (%) Primary school Secondary school Degree	38 (47.5) 29 (36.2) 13 (16.3)	9 (45) 8 (40) 3 (15)	31 (51.6) 21 (35) 8 (13.4)	0.47 0.62
Occupation, *n* (%) Working Unemployed	55 (68.7) 25 (31.3)	16 (80) 4 (20)	39 (65) 21 (35)	0.27
Duration pain (years), mean ± SD	2.8 ± 1.1	2.7 ± 1.9	2.8 ± 1.4	0.80
Vitamin D serum level (ng/mL), mean ± SD	22.8 ± 5.1	22.3 ± 4.2	23.1 ± 3.8	0.43
NRS, mean ± SD	7.4 ± 0.8	7.6 ± 1.1	7.4 ± 0.8	0.38
FIQ scale, mean ± SD	55.7 ± 7.7	58.1 ± 9.2	54.8 ± 6.9	0.10
SF-12 scale, mean ± SD	25.7 ± 3.1	25.2 ± 3.5	25.9 ± 2.9	0.37

**Table 2:** Effects of vitamin D supplementation at 3 months in group A.

Characteristics	T0	T1	*p*-Value
NRS, mean ± SD	7.6 ± 1.1	5.9 ± 1.1	<0.05
FIQ scale, mean± SD	58.1 ± 9.2	45.3 ± 6.6	<0.05
Vitamin D serum level (ng/mL), mean ± SD	22.3 ± 4.2	31.1 ± 3.8	<0.05
SF-12 scale, mean ± SD	25.2 ± 3.5	28.9 ± 2.7	<0.05

**Table 3:** Effects of vitamin D supplementation at 6 months in group A.

Characteristics	T0	T2	*p*-Value
NRS, mean ± SD	7.6 ± 1.1	5.7 ± 0.9	<0.05
FIQ scale, mean ± SD	58.1 ± 9.2	44.4 ± 5.9	<0.05
Vitamin D serum level (ng/mL), mean ± SD	22.3 ± 4.2	36.9 ± 4.1	<0.05
SF-12 scale, mean ± SD	25.2 ± 3.5	27.2 ± 3.6	0.08

**Table 4:** Effects of vitamin D supplementation at 3 months in group B.

Characteristics	T0	T1	*p*-Value
NRS, mean ± SD	7.4 ± 0.8	6.1 ± 1.3	<0.05
FIQ scale, mean ± SD	54.8 ± 6.9	43.1 ± 7	<0.05
Vitamin D serum levels (ng/mL), mean ± SD	23.1 ± 3.8	33.3 ± 3.3	<0.05
SF-12 scale, mean ± SD	25.9 ± 2.9	30.3 ± 2.5	<0.05

**Table 5:** Effects of vitamin D supplementation at 6 months in group B.

Characteristics	T0	T2	*p*-Value
NRS, mean ± SD	7.4 ± 0.8	5 ± 1.1	<0.05
FIQ scale, mean ± SD	54.8 ± 6.9	52.4 ± 8.1	0.08
Vitamin D serum levels (ng/mL), mean ± SD	23.1 ± 3.8	39.2 ± 2.9	<0.05
SF-12 scale, mean ± SD	25.9 ± 2.9	31.4 ± 2.4	<0.05

**Table 6:** Comparison between the T1-T0 (ΔT1-T0) and T2-T0 (ΔT2-T0) variations between the two groups at 3 months (T1) and at 6 months (T2).

Characteristics	ΔT1-T0	ΔT2-T0
NRS Group A Group B *p-value*	1.7 ± 0.8 1.3 ± 0.8 *0.03*	1.9 ± 0.8 2.4 ± 0.8 *<0.05*
FIQ scale Group A Group B *p-value*	12.8 ± 4.8 11.8 ± 2.7 *0.25*	13.7 ± 2.7 2.4 ± 7.9 *<0.05*
Vitamin D Group A Group B *p-value*	8.8 ± 2.7 10.2 ± 3.6 *0.12*	14.6 ± 3.7 16.1 ± 3.5 *0.10*
SF-12 scale Group A Group B *p-value*	3.7 ± 1.8 4.4 ± 1.8 *0.14*	2 ± 3.5 5.5 ± 2.3 *<0.05*

## Data Availability

Data used to support the findings of this study are available from the corresponding author upon request.
